# Structural performance of FRP composite bars reinforced rubberized concrete compressive members: Tests and numerical modeling

**DOI:** 10.1016/j.heliyon.2024.e26222

**Published:** 2024-02-15

**Authors:** Ali Raza, Khaled Mohamed Elhadi, Muhammad Abid, Ahmed Farouk Deifalla, Muhammad Sohail Jameel, Yasser Alashker

**Affiliations:** a Department of Civil Engineering, University of Engineering and Technology Taxila, 47050, Pakistan; b Department of Civil Engineering, College of Engineering, King Khalid University, PO Box 394, Abha, 61411, Saudi Arabia; c College of Aerospace and Civil Engineering, Harbin Engineering University, Harbin, 150001, China; d Structural Engineering Department, Faculty of Engineering and Technology, Future University in Egypt, New Cairo, 11845, Egypt; e Department of Transportation Engineering and Management, University of Engineering and Technology Lahore, 54890, Pakistan; f Structural Engineering Department, Faculty of Engineering, Zagazig University, Zagazig, Egypt

**Keywords:** Compressive elements, Rubberized concrete, GFRP bars, FEM, Axial stress

## Abstract

Waste tyre rubber has become an environmental and health concern that needs to be sustainably managed to avoid fire hazards and save natural resources. This research work aims to study the structural behavior of glass fiber reinforced polymer (glass-FRP) reinforced rubberized concrete (GRC) compressive elements under monotonic axial compression loads. Nine GRC circular compressive elements with different axial and crosswise reinforcement ratios were fabricated. All the elements were 300 mm in diameter and 1200 mm in height. A 3D nonlinear finite element equation (FEM) was suggested for the GRC compressive elements using a commercial package ABAQUS. A parametric study has been done to examine the effect of various parameters of GRC elements. The test outcomes revealed that the ductility of GRC elements ameliorated with the lessening in the spaces of glass-FRP ties. The addition of rubberized concrete improved the ductility of GRC elements. The damage to GRC elements occurred due to the vertical cracking along the height of the elements. The estimates of FEM were in close agreement with the test outcomes. The suggested empirical equation depending on the 600 test elements, which considered the lateral confinement effect of FRP ties, presented higher accuracy than previous equations.

## Highlights

• Nine GFRP-reinforced rubberized concrete columns were manufactured.
• 3D numerical simulations of all samples was performed.
• All tested samples failed in the upper and middle portion with improved ductility.
• Numerical and theoretical predictions presented close agreement with experiments.

## 1 Introduction

Nowadays, all over the world, waste tyre management has emerged as a critical health, aesthetic, and environmental concern. Such types of waste material have become the reasons for the potential environmental pollution including fire hazards, due to stockpiling, in addition to a suitable environment for the growing of mosquitos, mice, rats, etc. [1,2]. This area of sustainability became the spotlight of advanced research to manage waste rubber because up-to-date studies show that the quantity of waste rubber exceeds recycled rubber [3]. The utilization of rubber in concrete, called rubberized concrete, has become a sustainable solution for minimizing the waste of tyre rubber and saving the natural resources of aggregates [[4], [5], [6]]. Waste tyre rubber is an efficient structural material due to its high damping potential, high seismic resistance, high-energy dissipation capacity, and high impact resistance [[7], [8], [9], [10], [11], [12]].

The essential properties of steel reinforcement in reinforced concrete (RC) structural elements, such as stress and ductility, can be reduced because of the corrosive features of steel, leading to high maintenance costs and abridged structural behavior. Fiber-reinforced polymer (FRP) materials can replace steel reinforcement, which is a safe and possible solution. Because of their greater tensile stress, lower thermal conductivity, lower density, and greater resistance against corrosion, these composites are achieving genuine appropriateness and confidence [[13], [14], [15], [16]]. FRP bars could be used in corrosive and aggressive environments to minimize repairing expenses, improve service life, and reduce the total life-cycle cost of concrete structures [[17], [18], [19], [20], [21], [22], [23], [24], [25], [26]].

The mechanical efficacy of rubberized concrete has been well explored in previous studies by replacing the aggregates of concrete with rubber aggregates [[7], [8], [9], [10], [11], [12]]. Some of the previous studies concluded that the replacement of natural aggregates with rubber aggregates resulted in the minimization of the brittle behavior of concrete, making it more ductile and extending its plastic failure capacity by absorbing higher amounts of energy under tensile as well as compression loads [3,27,28]. They also concluded that the rubberized concrete element is capable of withstanding effective post-collapse loads and deflections without showing complete crumbling. The test testing on the flexural elements containing rubberized concrete depicted higher toughness values when compared with elements containing natural aggregates [29]. The slump values of rubberized concrete are decreased when the replacement ratio of rubber aggregates is enhanced, producing more air voids and reducing the unit weight of concrete [30]. Rubberized concrete shows higher ductility indices and lower brittleness indices associated with natural aggregate concrete [2]. A study on the explosive spalling of high-stress concrete containing crumb rubber showed that the rubberized concrete depicts a reduced risk of explosive spalling at elevated temperatures [31]. This can be ascribed to the water vapor existing through the channels produced due to burnt rubber aggregates.

When GRC compression elements are associated with steel-RC compression elements, the glass-FRP-RC slender elements presented larger lateral deflection and demonstrated greater ductility [[32], [33], [34], [35]]. Because steel and glass-FRP reinforcement are equally effective, the axial load-carrying capacity of glass-FRP reinforced compression elements is 7% lower [36]. Employing glass-FRP bars in compression elements is advantageous since their axial reinforcement performs better with concrete than their tensile reinforcement [37]. When glass-FRP bars are used in place of a comparable amount of steel reinforcement in concrete compression components, the axial capacity and bending stresses are reduced while the element ductility is marginally improved under various loading conditions [38]. Glass-FRP reinforced compression elements must have sufficient crosswise constriction to withstand ultimate loads equal to or greater than those of steel-RC compression elements in order to use glass-FRP bars [39]. Large GRC compression elements were damaged as a result of primary glass-FRP bars buckling at less transverse reinforcement (0.7% by volume), whereas enough transverse reinforcement (1.5% and 2.70% by volume) caused fractures to form as a result of crosswise confinement rupture and concrete core damage [40]. Depending on the examination of the impact of glass-FRP reinforcement, many methods for evaluating the axial loading stress of GRC compression elements have been presented [[40], [41], [42]]. Twelve circular-shaped GRC compression elements were studied, and the outcomes showed that the moment and main loading capacity of the GRC elements were much lower than their steel-RC equivalents [38,[43], [44], [45]]. To improve the axial strength, axial strain, and confining efficacy of GRC hollow compression elements, the number, and diameter of the main glass-FRP bars were amplified [46,47].

Finite element equations (FEM) are currently the focus of engineering research. These are regarded to be a much more efficient tool for monitoring the damaging behavior of FRPs and the bonding characteristic of concrete and FRPs in the least amount of time and cost [[48], [49], [50], [51], [52]]. Finite element analysis (FEA) was used to forecast the behavior of GRC compression elements with various slenderness ratios, and it was observed that the FEA predictions and test outcomes of failure modes and post-buckling behavior of the elements were highly correlated [53,54]. With solid modeling and estimation of the boundary conditions in FEA, empirical equations can accurately forecast the load-deformation curves and failure modes of GRC compression elements [18]. The complete load-deformation response, failure modes, and moment interaction diagrams were examined using an in-depth numerical study on GPC compression elements reinforced with glass-FRP bars, leading to the conclusion that the FEM accurately monitored the structural response of such elements by considering the elastic and plastic behavior of GPC [54]. The close estimate of FRP-RC geopolymer concrete compression elements with their empirical observations for the load-deflection graphs, failure modes, cracking patterns, and post-peak collapse behavior was reported using a modified concrete damage plasticity equation for FEA simulations in ABAQUS [55].

### 1.1 Research scope and significance

To the authors' knowledge and according to the extensive literature review, the axial compression behavior of glass-FRP-RC rubberized concrete (GRC) compression elements has not been investigated. Although rubberized concrete composites generally have an abridged compression stress that may limit their applications in certain structural engineering areas, it has a number of desired features, such as higher impact resistance, higher toughness, and lower density compared to conventional concrete. Landfilling of tyres outcomes in a severe environmental risk. Waste tyre disposal areas contribute to biodiversity loss such as tyres containing hazardous and soluble components. Recycling is one of the most significant waste decrease techniques; however, recycling discarded tyres is especially difficult because of their high production rates and non-biodegradability. Integrating waste tyres as a fractional replacement for coarse aggregate in concrete is one approach to drop the volume of waste tyres in the environment. Therefore, in the present investigation, experiments and three-dimensional nonlinear FEA under monotonic axial compression are used to investigate the behavior of GRC compression elements. The effects of axial glass-FRP bars and the gap of crosswise glass-FRP hoops on the axial load-carrying stress (LCS), axial deformation, damaging behavior, and cracking pattern of GRC compression elements were explored. Using a modified damaged plastic equation in ABAQUS, a nonlinear FEA equation was suggested that took into consideration the lateral confinement effect. A thorough investigation into the impact of various GRC compression member characteristics was conducted. The suggested compression member is a suitable structural element in terms of budget, resource requirements, and long-term effect on the environment. This study's findings may be useful to structural engineers when studying and fabricating sustainable concrete compression elements.

## 2 Test program

### 2.1 Materials

#### 2.1.1 Rubberized concrete

Developing an improved quality rubberized concrete is the crucial part of the present study because it consists of defining the suitable amounts of ingredients and a suitable amount of water following the proper mixing procedure. The mix design employed by a previous study Ref. [56] was implemented to fabricate the rubberized concrete. The natural coarse aggregates were substituted with waste tyre rubber aggregates in the manufacture of GRC. The Portland cement following the specifications of AS 3972 [57] was used as a binder. Table 1 shows the chemical composition of cement. The largest size of rubber aggregates was 19 mm. The natural coarse aggregates consisted of the largest size of 19 mm crushed rocks. The size of sand particles was in the range of 0.2–6 mm.

**Table 1:** Chemical composition of cement.

Compound	Percentage (%)	Compound	Percentage (%)
CaO	64	SiO_2_	20.5
Al_2_O_3_	5.3	Fe_2_O_3_	3.0
SO_3_	2.1	MgO	1.3
Na_2_O	0.5	LOI	1.8

**Fig. 1:**
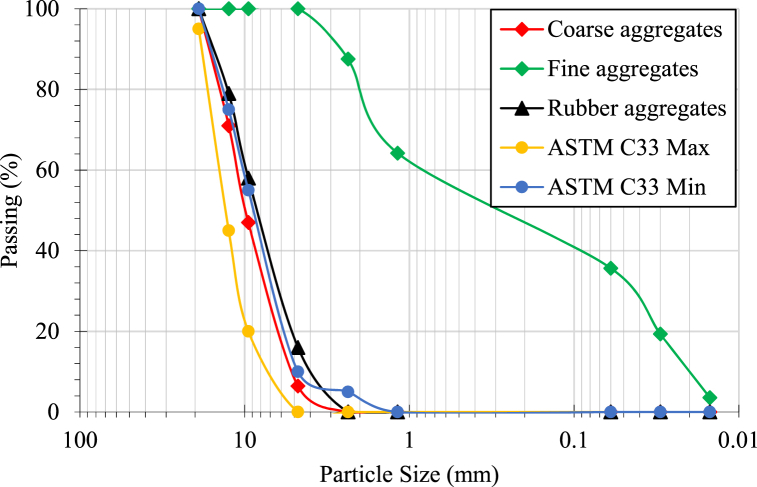
Granulometric analysis of natural coarse aggregates, sand, and rubber aggregates and ASTM minimum and largest size requirements for coarse aggregates.

The rubberized aggregates having the largest size of 10 mm were manufactured using scrap tyre rubbers that were employed to substitute the natural coarse aggregates by 20%. The sieve analysis outcomes of the natural coarse aggregates, sand, and rubber aggregates are shown in Fig. 1. The rubber aggregates were thoroughly washed with water to remove the dust particles that adhered to their surface to ensure good bonding among the cement and rubber aggregates. The dust particles were attached to the surface of the rubber aggregates during their fabrication process using mechanical grinding at room temperature. For the prevention of floating rubberized aggregates in concrete, they were pre-soaked in water for 24 h. Then the rubber aggregates were dried to obtain a saturated surface dry condition. The absorbed quantity of water by the rubberized aggregates was calculated and later on, subtracted from the required water content for fabricating the concrete mix. Various properties of coarse rubberized aggregate, natural coarse aggregates, and fine aggregates are presented in Table 2.

**Table 2:** Properties of coarse rubberized aggregate, natural coarse aggregates, and fine aggregates.

Property	Fine aggregates	Rubberized coarse aggregate	Natural coarse aggregates
Water absorption (%)	1.98	1.5	2.1
Specific gravity	2.67	1.15	2.69
Fineness modulus	2.42	4.58	–
Dry density (kg/m^3^)	1620	640	1488
Minimum size (mm)	0.2	4.75	1
The largest size (mm)	6	19	19

Table 3 shows the ingredients of GRC. The mixing procedure employed by a previous study Ref. [56] consisted of the following steps: (I) 1-min mixing of dry natural aggregates, (II) addition of 10% water and rubber aggregates (20% replacement of natural coarse aggregates) to already mixed aggregates, (III) addition and 1-min mixing of cement, (IV) addition and 1-min mixing of half of the remaining water (up to 90% of water), (V) at the end, addition and 1-min final mixing of the remaining water. A superplasticizer Viscocrete-3200 (250–300 ml per cubic meter of concrete) was used to secure a homogeneous and workable rubberized concrete mix. The slump test presented a slump value of 150 mm. Three cylinders having a size of 150 mm × 300 mm were fabricated to investigate the compression stress of rubberized concrete. The compression stress of rubberized concrete at 28 days was 28 MPa having a standard deviation of 2.1 MPa.

**Table 3:** Constituents of rubberized concrete.

Material	Quantity	Material	Quantity
Sand	843 kg/m^3^	Water	205 kg/m^3^
Natural coarse aggregates	704 kg/m^3^	Water to cement ratio	0.48
Cement	426 kg/m^3^	Superplasticizer	250–300 ml/m^3^
Rubberized aggregates	202 kg/m^3^	–	–

#### 2.1.2 Glass-FRP bars

The 9.5 mm diameter glass-FRP hoops provided crosswise reinforcement and the 12.7 mm diameter glass-FRP bars provided axial reinforcement. Glass-FRP hoops have a splice length of 65 mm. The painted glass-FRP bars and ligatures were produced from E-glass fibers impregnated in fillers, additives, and thermosetting vinyl ester resin accounting for 80% of the volumetric quantity. The B.2 test, as recommended by ACI 440.3R [58], was used to determine the stress attributes of the glass-FRP bars. Table 4 lists the parameters of glass-FRP bars.

**Table 4:** Parameters of glass-FRP bars.

Bar number	Diameter (mm)	Area (mm^2^)	Ultimate stress (MPa)	Young's modulus (MPa)	Ultimate strain (%)
#3	9.5	70.5	765	48,000	2.123
#4	12.7	126.8	830	50,000	2.035

### 2.2 Elements fabrication

**Fig. 2:**
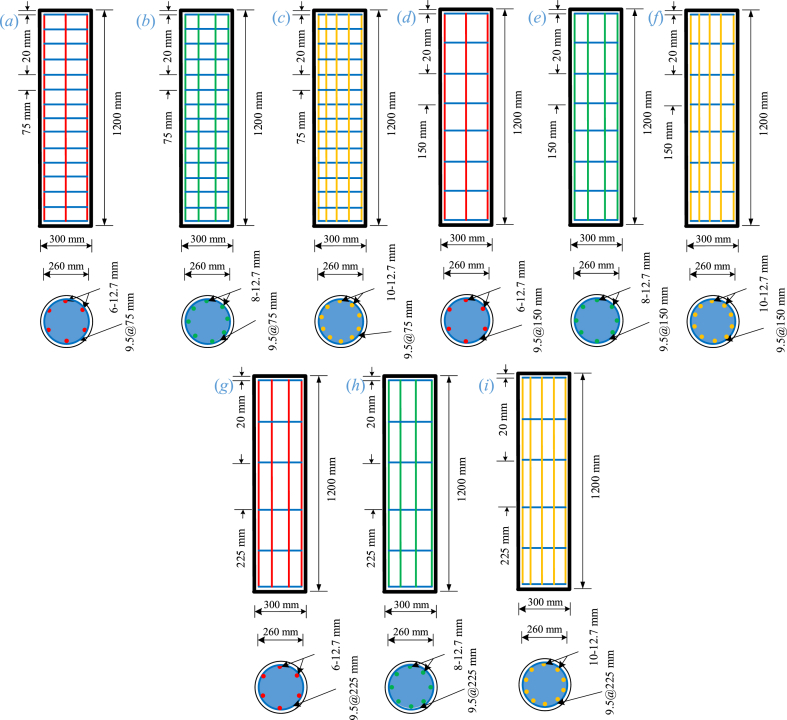
Dimensions and drawings of fabricated elements (*a*) GRC6-75 (*b*) GRC8-75 (*c*) GRC10-75 (*d*) GRC6-150 (*e*) GRC8-150 (*f*) GRC10-150 (*g*) GRC6-225 (*h*) GRC8-225 (*i*) GRC10-225.

The impact of different numbers of axial glass-FRP bars and glass-FRP tie spaces on the LCS of compression elements was studied in this work. Nine circular GRC compression elements were made and examined. The elements had a height of 1200 mm and a diameter of 300 mm, which are classified as full-size compression elements. Three different types of elements were created. Each set included three elements, each with 6, 8, or 10 axial glass-FRP bars of 12.7 mm in diameter. The vertical spaces of glass-FRP hoops (9.5 mm thick) were 75 mm, 150 mm, and 225 mm in the first, second, and third elements of each set, separately. In the first, second, and third sets, the axial glass-FRP reinforcement ratios were 1.43%, 1.91%, and 2.38%, respectively. In low seismicity zones, these reinforcement ratios may be recommended [55]. Such size and reinforcement of the fabricated elements were selected so that they could be easily tested and placed in the compression machine available in the laboratory. The volumetric ratios of the crosswise glass-FRP hoops for the first, second, and third elements of each set were 1.42%, 0.71%, and 0.50%, respectively. The spaces of crosswise glass-FRP hoops were chosen to ensure the elastic failure of glass-FRP axial bars [59]. For all test elements, the concrete cover was retained at 20 mm. Fig. 2 shows the geometrical and sectional characteristics of fabricated elements.

The elements' geometry and testing characteristics are listed in Table 5. As a formwork for the elements, a 10 mm thick PVC tube with an inside diameter of 300 mm was used. Spacers were employed to provide the concrete cover of 20 mm.

**Table 5:** Details of test elements.

Compression member ID	Axial bars			Crosswise bars		
	Diameter (mm)	No. of bars	Reinforcement ratio (%)	Diameter (mm)	Pitch (mm)	Volumetric ratio (%)
GRC6-75	12.7	6	1.43	9.5	75	1.42
GRC6-150					150	0.71
GRC6-225					225	0.50
GRC8-75	12.7	8	1.91	9.5	75	1.42
GRC8-150					150	0.71
GRC8-225					225	0.50
GRC10-75	12.7	10	2.38	9.5	75	1.42
GRC10-150					150	0.71
GRC10-225					225	0.50

### 2.3 Testing assembly

**Fig. 3:**
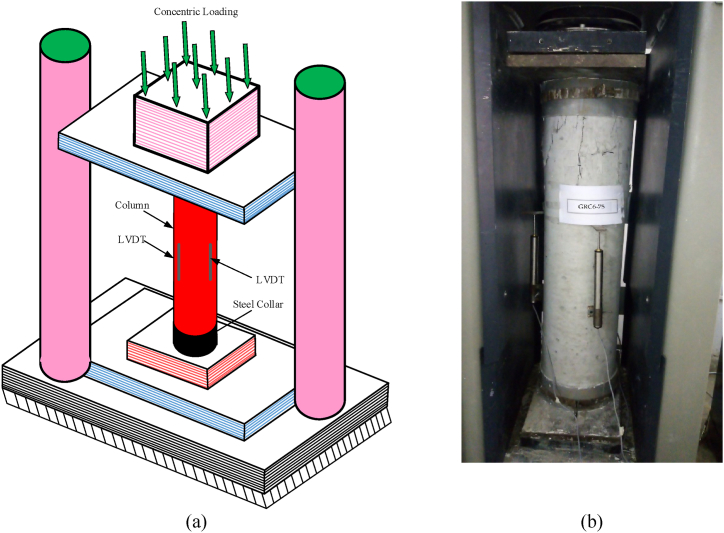
Testing setup of elements (a) schematic diagram (b) experimental setup.

During the test testing of elements, to minimize end crushing and apply a consistently distributed stress across the cross-section of the element, the bottom and top surfaces of elements were smoothed with plaster after adding steel collars having dimensions of 100 mm in diameter and 10 mm in thickness, as shown in Fig. 3. To remove any misalignment, the elements were subjected to a preload of 100 kN using a 5000 kN compression testing machine by load control approach. The rate of loading was 0.8 kN/s for the preloading. The elements were released at the same rate and subsequently loaded at a rate of 0.3 mm/min using a displacement control approach. Three LVDTs were installed and spaced 120° apart to measure the axial deformation of the compression elements. A P3 box attached to the testing equipment was used to record axial load and axial deformation values.

## 3 FEA modeling of GRC compression elements

**Fig. 4:**
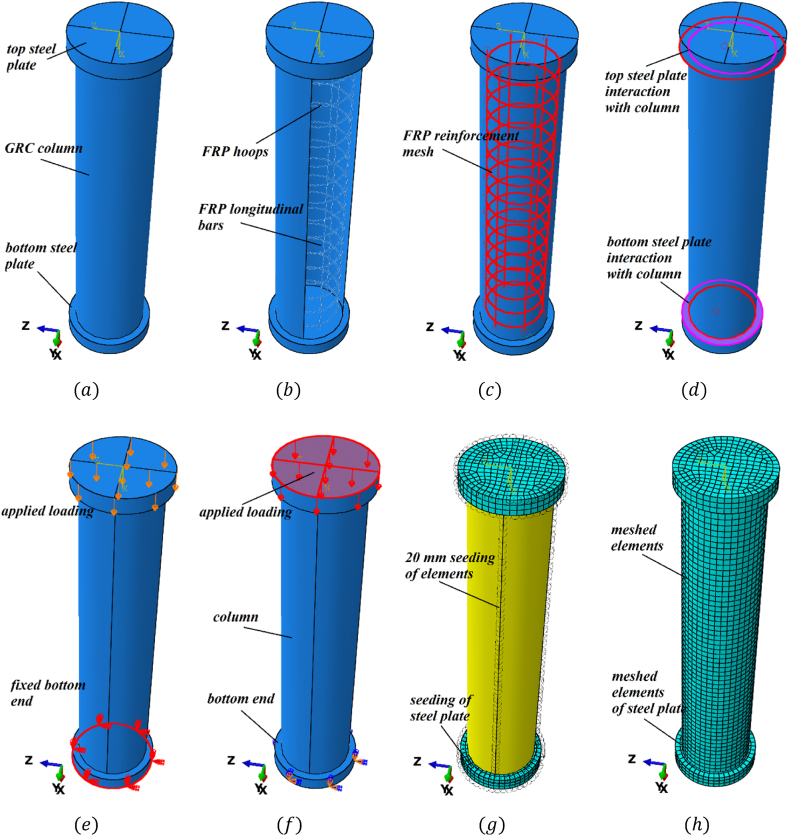
Simulated control equation (a) assembly (b) FRP reinforcement (c) embedded region for FRP (d) interaction of steel plate with compression member (e) fixed bottom (f) applied loading (g) seeding of elements (h) meshed elements.

The FEA of GRC compression elements was performed using the commercial tool ABAQUS 6.14. The suggested FEM took into account the elements' initial stiffness, failure mechanism, crack propagation, ultimate stress, and post-cracking stiffness. The test data of a control element (GRC6-75) was used as a benchmark for the FEM calculations. Deformable truss elements were employed in the modeling of glass-FRP bars, while deformable stress elements were used in the modeling of GRC. Glass-FRP bars were used to describe the linear elastic equation, whereas GRC was defined using a modified version of the well-known concrete damage plasticity (CDP) equation by considering the compression, plastic, and tensile behavior. All supports were constrained at the base of the element, and a uniformly distributed load was applied to the top surface using a displacement control method. In ABAQUS, the “embedded region” requirement was used to define the link between the glass-FRP bars and the GRC [18]. For the implementation of boundary conditions identical to test boundary conditions, the steel plates (50 mm thick) were attached to the top and bottom surfaces of the element using the “tie” constraint. Fig. 4(a–h) shows the simulations of the FEA models of the elements.

### 3.1 Simulation of GRC

The elastic response of GRC can be specified in ABAQUS using two factors: Poisson's ratio, which was set to 0.15 [60], and the elastic modulus, which was set to $2707\sqrt{f_{co}^{\prime }}+5300$ [60]. where, $f_{co}^{\prime }$ is the compression stress of GRC. The modified CDP equation was used to define the plastic, compression, and tensile behaviour of GRC. This equation can accurately simulate this behavior by addressing the combination of isotropic compression plasticity, isotropic tensile plasticity, and isotropic damaging elasticity of concrete [61,62].

#### 3.1.1 Plastic behavior

For the simulations of rubberized concrete, plastic behavior is also important to be accurately defined. For the understanding of the plasticity of concrete, the CDP equation takes into account five different variables: (1) the eccentricity (e), (2) the viscosity parameter (μ), (3) the ratio of biaxial to uniaxial compression yield stresses ($\sigma_{bo}/\sigma_{co}$), (4) the shape factor of the yielding surface ($K_{c}$), and (5) the dilation angle of GRC (ψ). Except for the parameter, which was discussed in the validation portion of this work, all of these variables were calibrated for different indices and showed no impact on the load-deformation behavior of the GRC control equation.

#### 3.1.2 Compression behavior

**Fig. 5:**
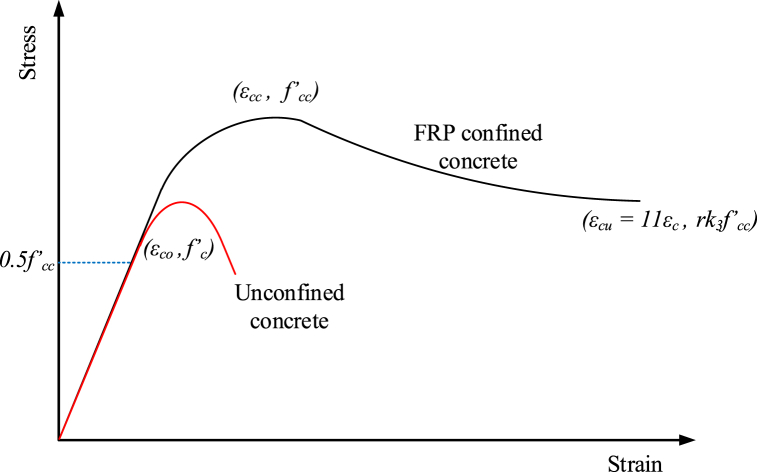
Stress-strain behavior for confined and unconfined GRC.

As presented in Fig. 5, the lateral confinement provided by glass-FRP improves the LCS and ductility of concrete [63]. When compared to unconfined concrete, the compression stress (CS) of the concrete improves, and the stress decrease at peak load is more gradual. Constrained concrete has an elastic efficacy of up to 50% of peak confined stress. As a result, the equations suggested by Afifi et al. [64] for the compression stress and strain of confined GRC, as represented by Eqs. (1), (2), were employed to account for the confinement enhancement effect due to glass-FRP connections.

(1) $$\frac{f_{cc}^{\prime }}{f_{co}^{\prime }}=1.0+4.547{\left(\frac{f_{le}}{f_{co}^{\prime }}\right)}^{0.723}$$

(2) $$\frac{\varepsilon_{cc}^{\prime }}{\varepsilon_{co}^{\prime }}=1.0+\left(\frac{0.024}{\varepsilon_{co}^{\prime }}\right){\left(\frac{f_{le}}{f_{co}^{\prime }}\right)}^{0.907}$$

where $f_{le}$ represents the significant confinement stress provided by the FRP ties which can be calculated from Eq. (3) [65].

(3) $$f_{le}=\frac{2E_{f}\varepsilon_{h,rup}t}{D}$$

Where $E_{f}$ is the lateral tie's elastic modulus, $\varepsilon_{h,rup}$ is the lateral tie's hoop rupture strains, and 't' is the lateral tie's thickness. Both the stress and stiffness of concrete are reduced after cracks emerge and propagate, and they cannot be regained. According to the notions of damage accumulation mechanics [66], the presence of shrinkage cracks and cracks created after the application of the load lessens the stress and stiffness of concrete. According to the concept of elastoplasticity assumption, the total strain (ε) can be separated into two portions: elastic strain ($\varepsilon^{el}$) and plastic strain ($\varepsilon^{pl}$) of concrete as reported by Eq. (4).

(4) $$\varepsilon =\varepsilon^{el}+\varepsilon^{pl}$$

**Fig. 6:**
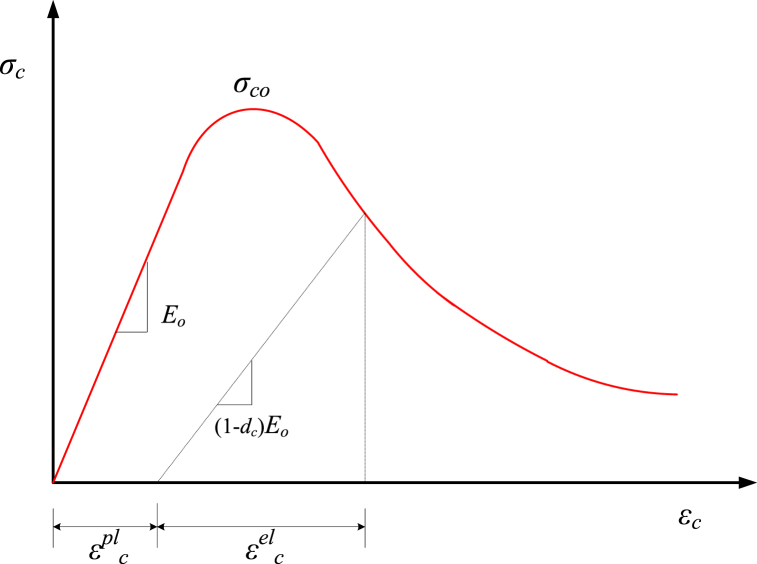
Compression stress-strain behavior of GRC.

Plasticity or damage can be used to equation concrete nonlinearity independently or in combination to simulate concrete nonlinearity. The degradation of stiffness escalates as the strain of concrete upsurges, inferring that the strain components ($\varepsilon^{el}$ and $\varepsilon^{pl}$) impact the evolution of concrete damage [67]. In the CDP equation, the uniaxial compression damage variable ($d_{c}$) and the uniaxial tensile damage parameter ($d_{t}$) are used to simulate GRC damage. By considering Fig. 6, the compression stress of GRC ($\sigma_{c}$) can be interpreted as represented by Eq. (5):

(5) $$\sigma_{c}=\left(1-d_{c}\right)E_{o}\left(\varepsilon_{c}-\varepsilon_{c}^{pl}\right)$$

where $E_{o}$ is the modulus of elasticity of GRC, $\varepsilon_{c}$ is the compression strain of GRC, and $\varepsilon_{c}^{pl}$ is the plastic portion of the compression strain of GRC. The factor $d_{c}$ is defined by Eq. (6) [68].

(6) dc=1e−1mc−1(e−εc,normin/mc−1)

where $m_{c}$ is the compression collapse evolution speed controlling parameter having an index of 0.05 [69], $\varepsilon_{c,norm}^{in}$ is the standardized compression plasticity strain of GRC that is interpreted by the ratio of inelastic strain to the standardized plasticity strain with an index of 0.033 [69].

#### 3.1.3 Tensile behavior

**Fig. 7:**
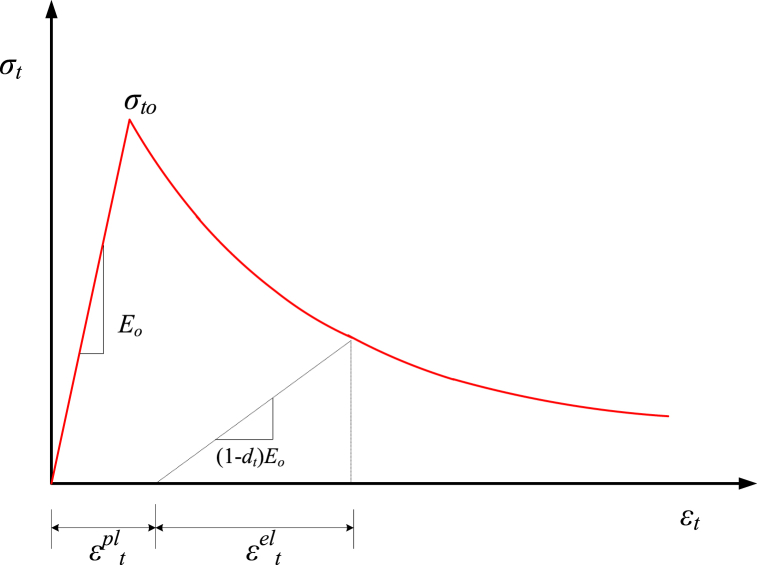
Tensile stress-strain behavior of GRC.

The post-cracking behavior of GRC compression elements is elaborated by the strain-hardening and strain-softening parts of the stress-strain graph. The pre-peak behavior during plastic flow is related to strain-hardening, while the post-peak behavior during concrete stress and stiffness degradation is connected to strain-softening [70]. Fig. 7 shows the tensile behavior of GRC, the tensile stress of GRC ($\sigma_{t}$) can be defined as reported by Eq. (7).

(7) $$\sigma_{t}=\left(1-d_{t}\right)E_{o}\left(\varepsilon_{t}-\varepsilon_{t}^{pl}\right)$$

where $\varepsilon_{t}$ is the tensile strain of GRC and $\varepsilon_{t}^{pl}$ is the plastic portion of the tensile strain of GRC. Wang and Chen [68] suggested an expression for calculating $d_{t}$ as represented by Eq. (8).

(8) dt=1e−1mt−1(e−εt,normck/mt−1)

In this equation, $m_{t}$ is the tensile collapse evolution speed governing factor having a value of 0.05 [69], $\varepsilon_{t,norm}^{in}$ is the standardized tensile plasticity strain of GRC that is interpreted by the ratio of inelastic tensile strain to the standardized tensile plasticity strain with a value of 0.0033 [69].

### 3.2 Modeling of Glass-FRP bars

**Fig. 8:**
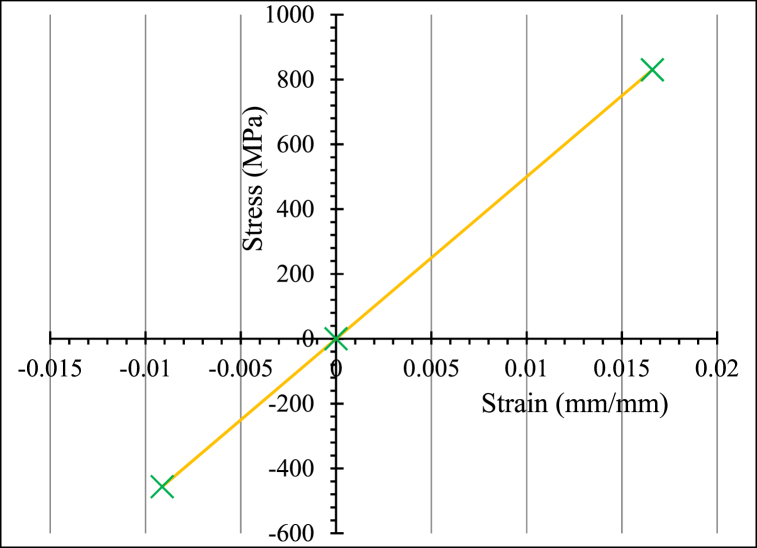
Linear elastic behavior of glass-FRP bars.

Glass-FRP bars' geometry was simulated using 3D truss components. As indicated in Table 4, FEM was used to equation different geometrical and mechanical reinforcing aspects. The Poisson's ratio of the glass-FRP bars is 0.25 [54]. To simulate the structural efficacy of glass-FRP bars, a linear elastic equation is considered [18,54,71] as shown in Fig. 8. The CS of glass-FRP bars is 55% of their tensile stress [72].

### 3.3 Validation analysis and mesh sensitivity

The load-deformation curve of the reference element GRC6-75 was matched to the forecasts of FEM during the calibration work. The validation for the boundary conditions showed a good agreement with the test outcomes when the element was restrained at one side and permitted for all degrees of freedom with the applied load in compression at the other side. The CDP equation's plasticity parameters that will be included in ABAQUS were all calibrated for various indices. For instance, the parameter Kc's value falls between 0.64 and 0.80 [73]. A score of 0.70 indicates a close correlation with the test results when the concrete is subjected to considerable hydrostatic forces [67]. For concrete, 0.1 can be considered for the parameter ‘e’ [67]. The dilation angle is a concrete material variable, and a value of 40° improves the efficacy of concrete compression elements [74]. Moreover, the parameter $\sigma_{bo}/\sigma_{co}$ can be calculated for GRC using Eq. (9):

(9) $$\frac{\sigma_{bo}}{\sigma_{co}}=1.5{f_{co}^{\prime }}^{-0.075}$$

No discernible variations were seen for varying levels of these limitations. Therefore, as shown in Table 6, the default settings for these limitations were used in the current study. The convergence of finite element simulations is limited by the viscosity parameter during the strain localization phenomenon. If its amount is low, the convergence rate will be improved [75]. The validation process for the factor revealed that 0.0025 provided superior efficacy for the load-deformation response of the control equation.

**Table 6:** Values of plasticity parameters and mesh size (MS) for GRC compression elements in ABAQUS.

Parameter	Quantity	Parameter	Quantity
ψ	40^o^	μ	0.0025
$\sigma_{bo}/\sigma_{co}$	1.15	$K_{c}$	0.70
*e*	0.1	MS	20 mm

The sensitivity of the reference equation's load-deformation response was also investigated for various element sizes (mesh sizes). Uneven mesh sizes are to blame for the inconsistent transmission of loads and strains in the elements. Mesh sensitivity has to be researched as a result. We looked at mesh sizes between 10 mm and 60 mm. Using a 20 mm mesh size, remarkably close matches to test findings were produced. For the different forms of GRC and glass-FRP reinforcement, sensitivity analyses were included in the calibration technique to make sure that the FEM calculations and testing data were closely correlated. While the GRC was investigated for hexahedral, triangular, and tetrahedral components, the glass-FRP bars were measured for truss elements. GRC and glass-FRP bars with various element types did not exhibit any appreciable differences. The study found that the FEM used T3D8R for GRC and T3D2 for glass-FRP bars [18,62,67].

## 4 Discussion and comparison of outcomes

### 4.1 Load-deformation efficacy

The suggested FEM successfully predicted the fracture patterns, elastic behavior, and post-peak collapse behavior of GRC6-75. The measurements revealed divergences for the largest LCS and associated axial deformation in the control equation of 2.81% and 8.48%. (GRC6-75). The minor discrepancies shown by the suggested FEM could be ascribed to variations in test and FEM conditions, such as material stress, the discrepancy in glass-FRP reinforcement features, boundary conditions, defects in element manufacture, initial geometric imperfections, and testing apparatus accuracy. The assumptions of a flawless connection between the GRC and the glass-FRP reinforcement may be to blame for the discrepancies. On the other hand, the suggested FEM showed a significant association with the outcomes of the experiment. Table 7 shows the test and FEM outcomes for the peak LCS, the peak LCS deformations, and the percent differences between test and FEM outcomes. The GRC8-75 equation provided the highest difference between test observations and FEM projections for the peak LCS, with an inaccuracy of 5.73%. The element GRC6-75 provided the highest difference in the axial deformation at peak LCS, with an inaccuracy of 8.48%. These relatively high divergences may have happened because the finite element simulations did not account for the elements' original geometric flaws. For GRC compression elements, the average difference between test and FEA values was 3.58% for peak LCS and 4.01% for axial deformation at peak LCS. The peak load-carrying capacity of GRC compression elements was underestimated in the majority of FEM studies.

**Table 7:** Summary of outcomes.

Compression member ID	Test outcomes		FEA outcomes		%age difference in P_u_	%age difference in deformation at P_u_	Ductility index	Average difference for P_u_	Average difference for deformation at P_u_
	P_u_ (kN)	Axial deformation at P_u_ (mm)	P_u_ (kN)	Axial deformation at P_u_ (mm)					
GRC6-75	1818	3.89	1767	3.56	2.81	8.48	2.23	1.61	5.21
GRC6-150	1673	3.73	1655	3.77	1.08	1.07	1.98		
GRC6-225	1585	4.12	1570	4.37	0.95	6.07	1.78		
GRC8-75	1955	3.9	1843	3.76	5.73	3.59	2.16	4.94	3.27
GRC8-150	1884	3.35	1810	3.4	3.93	1.49	1.67		
GRC8-225	1728	4.03	1817	3.84	5.15	4.71	1.43		
GRC10-75	1885	4.36	1809	4.43	4.03	1.61	1.83	4.19	3.55
GRC10-150	1746	4.27	1681	4.4	3.72	3.04	1.51		
GRC10-225	1637	3.5	1558	3.71	4.83	6.00	1.27		

**Fig. 9:**
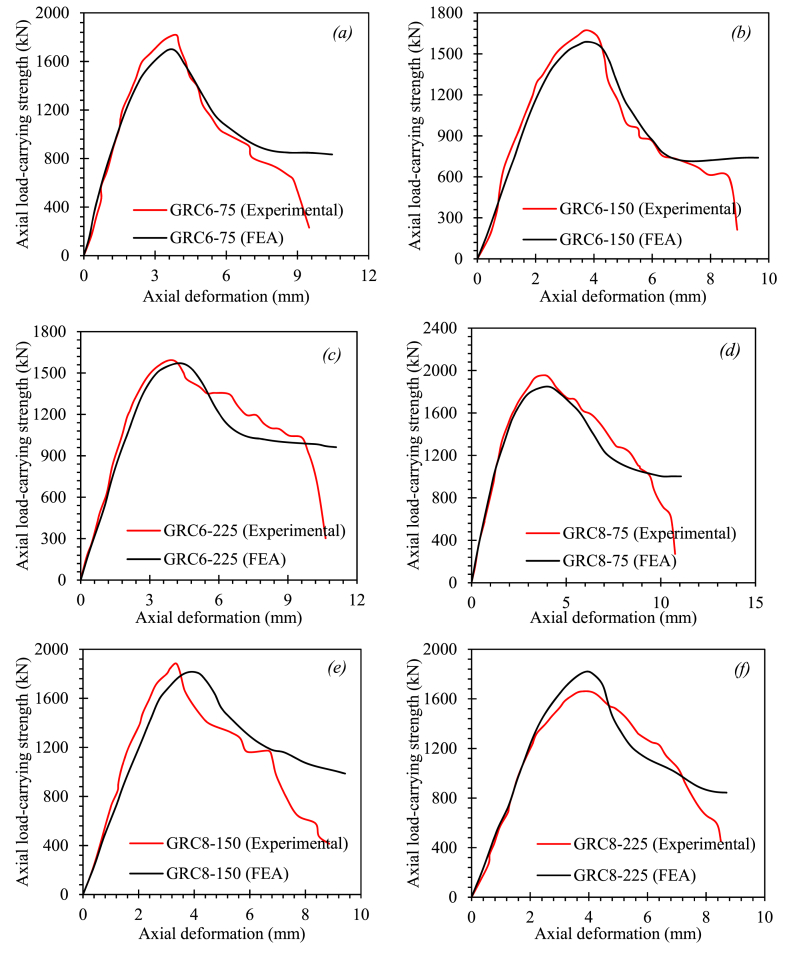

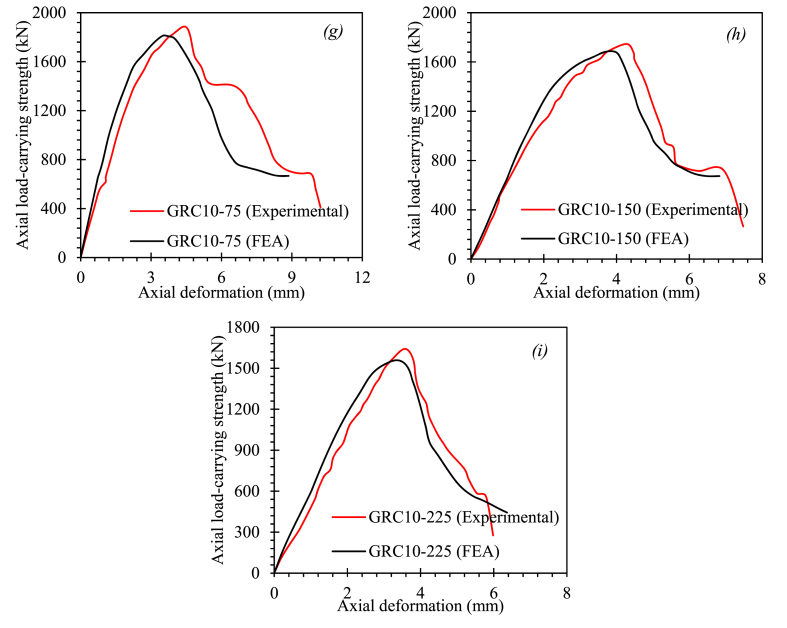
Comparison between FEM and test load-deformation curves of GRC compression elements (*a*) GRC6-75 (*b*) GRC6-150 (*c*) GRC6-225 (*d*) GRC8-75 (*e*) GRC8-150 (*f*) GRC8-225 (*g*) GRC10-75 (*h*) GRC10-150 (*i*) GRC10-225

Fig. 9 shows a comparison of the test and computed complete load-deformation graphs of GRC compression elements. With minor inconsistencies, the suggested FEM calculated the elastic and inelastic response of GRC compression elements. The elements GRC10-75 and GRC10-225 had stiffer outcomes in both the elastic and inelastic ranges, while all other FEM equations underestimated the stiffness behavior in the elastic response of elements. The modest differences in post-peak collapse behavior of the GRC compression elements between test measurements and FEM outcomes reveal the complicated damaging, cracking, and degradation behavior of GRC, as well as the bonding interaction between glass-FRP bars and GRC, need to be improved further.

### 4.2 Failure modes and damage

**Fig. 10:**
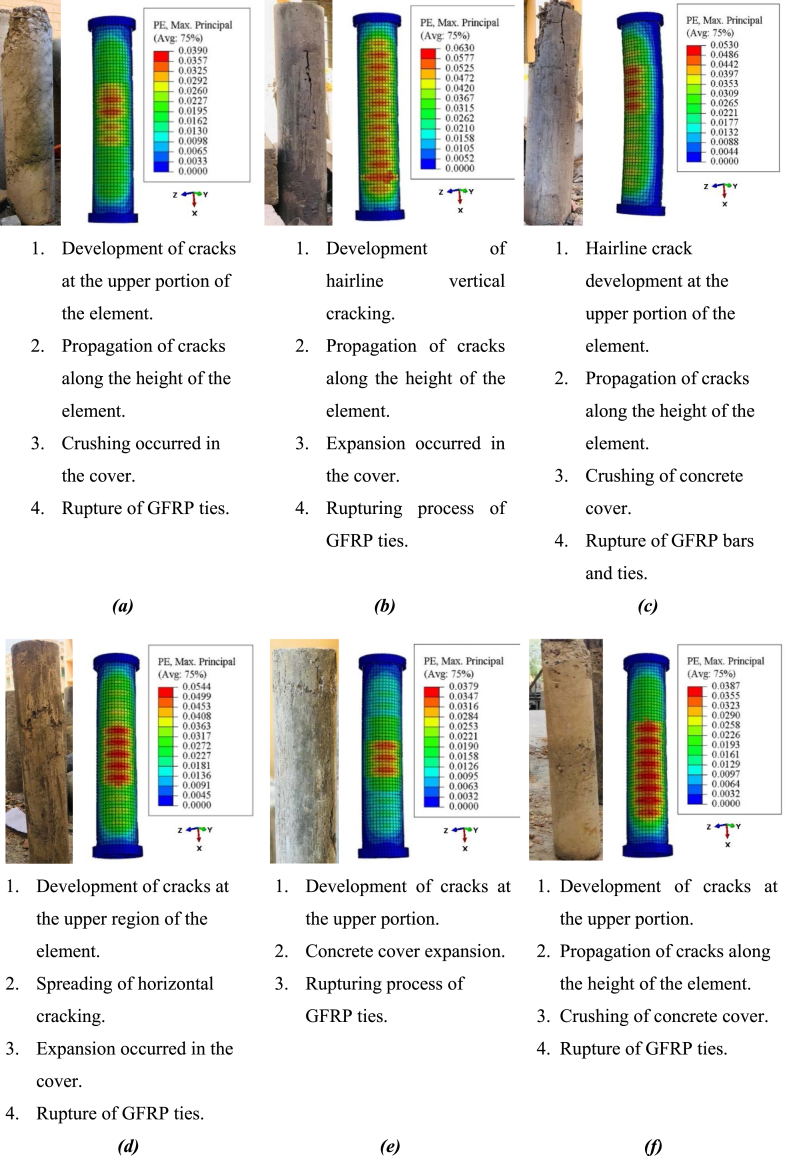

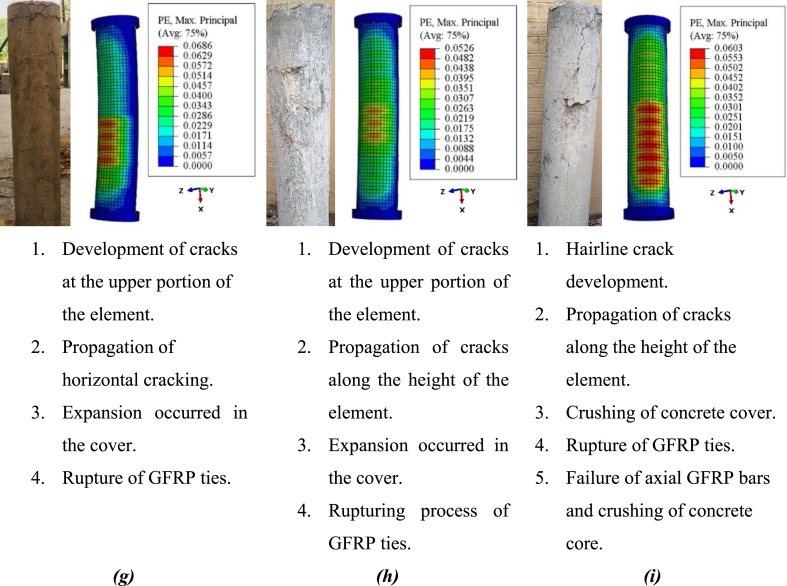
Failure modes of GRC elements (*a*) GRC6-75 (*b*) GRC6-150 (*c*) GRC6-225 (*d*) GRC8-75 (*e*) GRC8-150 (*f*) GRC8-225 (*g*) GRC10-75 (*h*) GRC10-150 (*i*) GRC10-225

The buckling of glass-FRP bars and the rupturing of lateral glass-FRP ties caused all of the GRC elements to fail in the middle region. After being subjected to axial compression load, the elements performed elastically up to about 80% of their largest load. The confining result generated by the lateral glass-FRP links was not activated at that time. The GRC began cracking with a faint sound and a vertical hairline crack emerged in the elements when the load was enhanced further. The cracks propagated together with the height of the elements as the load was steadily enhanced up to the peak value, and the crack breadth amplified. The concrete cover began to crack, and glass-FRP ties were used to give crosswise confinement to the concrete core. At the final stage of this procedure, the test element represented the fracture of glass-FRP ties at roughly 75 percent of their largest value in the post-damage phase, the buckling and rupture of main glass-FRP bars, and the crushing of the contained GRC core. The damage quantification of FEM and test fracture patterns and failure modes are compared in Fig. 10. Cracks in GRC were predicted using the largest positive primary plastic strains in the FEM. As the alignment of the cracks is always normal for these strains, the cracking patterns may be correctly predicted [18,65,76,77]. For the capture of fracture patterns and failure modes of GRC compression elements, FEM provided satisfactory forecasts. When the reinforcement began to yield, the damage progression and spalling of the concrete cover began. As indicated by the test measurements, all of the elements failed either in the upper half or in the middle region. The GRC compression elements represented failure due to the development of vertical cracks first. However, the crushing failure of rubberized concrete was a controlling failure without converting it to debris. The controlling failure indicates the amplified deformability efficacy of rubberized concrete. The main reason behind the failure of rubberized concrete is the micro-cracking within the composite in the vertical direction of elements which outcomes in the controlled failure without a sudden crushing like normal concrete which shows an abrupt failure [78]. Therefore, the failure of rubberized concrete compression elements reinforced with FRP bars was ductile despite brittle FRP bar reinforcement.

### 4.3 Ductility efficacy

**Fig. 11:**
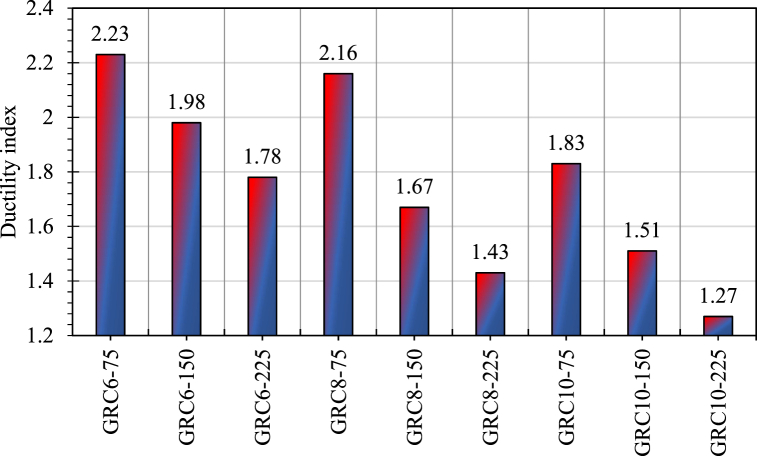
Ductility indices for various GRC elements.

Ductility refers to a structural element's capacity to absorb energy. As indicated by earlier studies, the ductility index for GRC compression elements was computed as the ratio of the area below the load-deformation graph up to 85 percent of peak load in collapse behavior and the area below the load-deformation graph up to 75 percent of peak load in pre-peak behavior [[79], [80], [81]]. The ductility indices for all GRC elements are shown in Fig. 11. The ductility indices were greater in the compression elements with lower stirrup spaces. The ductility indices of the elements GRC6-75, GRC8-75, and GRC10-75 were 2.23, 2.16, and 1.83, respectively. Due to its excellent containment, element GRC6-75 absorbed the most energy. The ductility index was strongly impacted by the gap of crosswise glass-FRP links, but the crosswise reinforcement ratio had also a significant effect on this parameter. For example, GRC6-75 had a 2.23 index, whereas GRC6-225 had a 1.78 index. GRC8-75 has a ductility value of 2.16, whereas GRC8-225 has a ductility index of 1.43. The mean ductility values for the GRC compression elements with a stirrup gap of 75 mm, 150 mm, and 225 mm were 2.07, 1.72, and 1.49, respectively. Due to their brittle character, elements with large reinforcement ratios had reduced ductility indices, providing minimal energy absorption. The well-restrained glass-FRP bars and the effective lateral confinement of the GRC core to absorb more energy may be responsible for the improved ductility of the GRC compression elements with lower stirrup spaces [81]. The controlling failure depicts the enhanced deformability behavior of rubberized concrete. The major cause behind the failure of rubberized concrete is the micro-cracking within the concrete which outcomes in controlled failure without sudden crushing like conventional concrete which shows an abrupt failure [78]. Thus, the failure of GRC compression elements was ductile despite brittle GFRP bars.

### 4.4 Effectiveness of axial bars

**Fig. 12:**
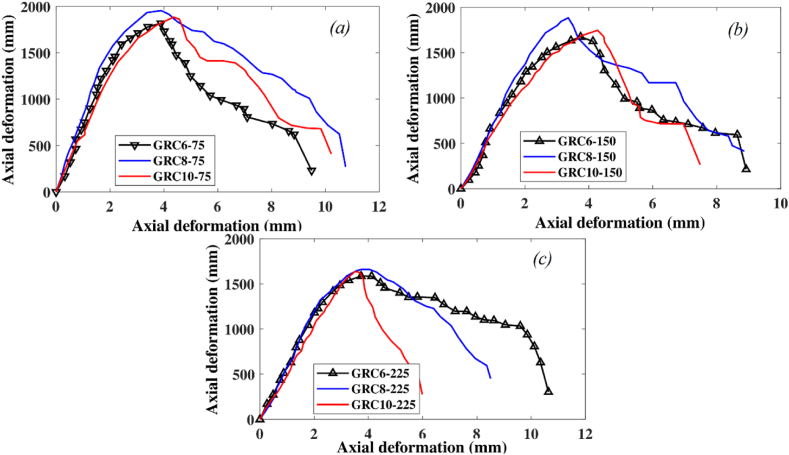
Impact of glass-FRP reinforcement ratio on the load-deformation response of GRC compression elements (a) 75 mm spacing of transverse reinforcement (b) 150 mm spacing of transverse reinforcement (c) 225 mm spacing of transverse reinforcement.

The effect of axial glass-FRP reinforcement on the full load-deformation efficacy of the GRC element is depicted in Fig. 12. Increasing the axial reinforcement ratio did not result in any substantial improvements in the LCS of elements other than making them more brittle. With increasing the number of glass-FRP bars from six (reinforcement ratio of 1.43%) to eight (reinforcement ratio of 1.91%) and 10, the LCS of GRC compression elements with 75 mm stirrup gap improved by 7% and 4%. By increasing the reinforcing ratio from 1.5% to 1.91% and 2.38%, the LCS of GRC compression elements with 150 mm gap spaces improved by 11% and 4%, respectively. When the reinforcement ratio was amplified from 1.43% to 1.91% or 2.38%, the LCS of GRC compression elements with a 225 mm stirrup gap improved by 8% and 3%, respectively. As a result, high axial reinforcement ratios did not result in a considerable axial capacity gain. Due to a higher effective ratio of axial bar stiffness to the effective GRC area, an element with a greater number of axial glass-FRP bars had a stronger axial stiffness response. At the peak load, the axial strain is relatively small which leads to a small contribution of FRP bars in the axial loading capacity of compression elements.

### 4.5 Effectiveness of stirrup spaces

**Fig. 13:**
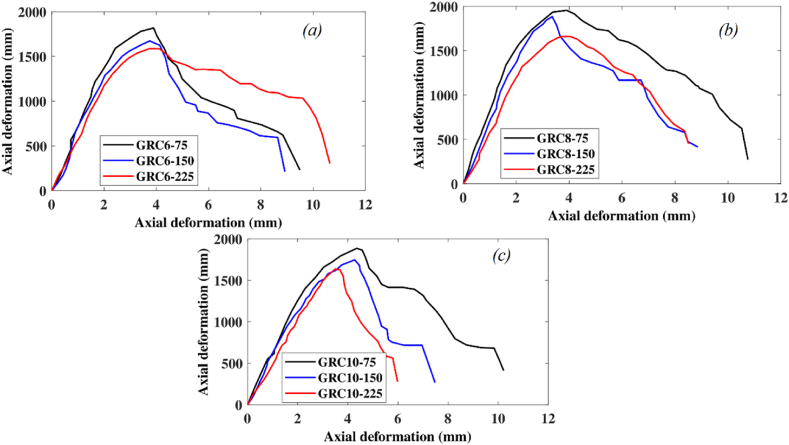
Impact of spaces of glass-FRP ties on the load-deformation response of GRC compression elements with (a) six main bars (b) eight main bars (c) ten main bars.

The effect of the vertical stirrup gap is depicted in Fig. 13. The LCS of GRC elements improved due to a lessening in the vertical spaces of glass-FRP links. For GRC elements with six axial bars, lowering the glass-FRP ties gap from 150 mm to 75 mm contributed to a percentage increase of 8%. For GRC elements with six axial bars, a percentage increase of 13% was recorded when the glass-FRP tie gap was lowered from 225 mm to 150 mm. For GRC elements with eight axial bars, lowering the glass-FRP tie gap from 150 mm to 75 mm donated to a 4% upsurge in percentage. For GRC elements with 8 axial bars, a percentage increase of 12% was recorded when the glass-FRP ties gap was lessened from 225 mm to 150 mm. Lowering the glass-FRP ties gap from 150 mm to 75 mm contributed to a 7% increase in percentage for GRC elements with 10 axial bars. For the GRC element with 10 axial bars, a percentage increase of 13% was detected when the glass-FRP tie gap was lessened from 225 mm to 150 mm. Due to the better-restrained glass-FRP bars and excellent confinement of the GRC core, the LCS of the GRC elements increases as the stirrup spaces decrease [81].

## 5 Parametric study

Following validation, the predicted FEM was utilized to simulate 600 GRC compression elements subjected to compression loads. Four factors of compression elements (a) axial FRP reinforcement ratio ($\rho_{l}$), (b) concrete CS ($f_{c}^{\prime }$), (c) the tensile stress of FRP bars ($f_{u}$), and (d) the diameter of compression elements (D) were modified for different ranges to examine their impact on the LCS of the compression elements as presented in Table 8. The primary goal of the numerical research work was to create a dataset of GRC compression elements with numerous geometrical and material variables so that the suggested empirical equation's estimates could be confirmed.

**Table 8:** Parameters considered for parametric study.

Variable	Fixed value	Investigated values
Concrete stress (MPa)	30	10, 15, 20, 25, 30, 35, 40, 45, 50, 55
Tensile stress (MPa)	850	700, 750, 800, 850, 900, 950, 1000, 1050, 1100, 1150
Diameter of compression member (mm)	200	150, 175, 200, 225, 250, 275, 300, 325, 350, 375
Reinforcement ratio (%)	1.94	0.97, 1.46, 1.94, 2.43, 2.92, 3.41, 3.89, 4.38, 4.86, 5.35

### 5.1 Effectiveness of diameter (*D*)

**Fig. 14:**
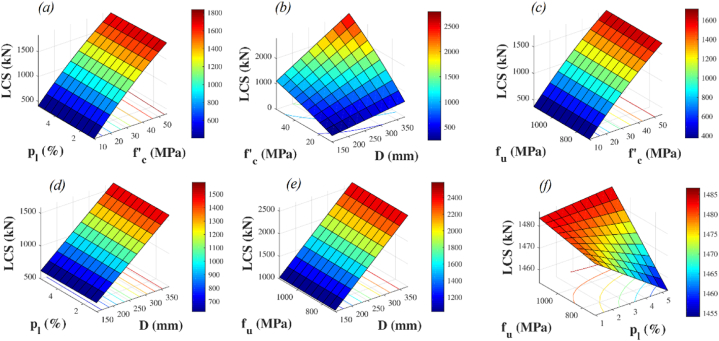
Detailed parametric investigation using FEM (a) effect of $\rho_{l}$ and $f_{c}^{\prime }$ on LCS (b) effect of D and $f_{c}^{\prime }$ on LCS (c) effect of $f_{u}$ and $f_{c}^{\prime }$ on LCS (d) effect of $\rho_{l}$ and D on LCS (e) effect of $f_{u}$ and D on LCS (f) effect of $f_{u}$ and $\rho_{l}$ on LCS.

The impact of D on the glass-FRP-RC elements' LCS is shown in Fig. 14. The analyzed indices were 150 mm–375 mm in length. The glass-FRP-RC elements' axial compression stress increased by 1040% when D grew from 150 to 375 mm, raising $f_{c}^{\prime }$ from 10 to 55 MPa while keeping $\rho_{l}$ at 1.94 percent and $f_{u}$ at 850 MPa. In a similar vein, increasing D from 150 to 375 mm caused a 150% rise in LCS when increasing $f_{u}$ from 700 to 1150 MPa.

#### 5.1.1 Effectiveness of concrete stress ($f_{c}^{\prime }$)

The effect of $f_{c}^{\prime }$ on the axial compression stress of compression elements is seen in Fig. 14. There is a notable 1040% improvement in the LCS as D climbs from 150 to 375 mm and $f_{c}^{\prime }$ rises from 10 to 55 MPa. By increasing $\rho_{l}$ from 0.97% to 5.35%, one can also improve $f_{c}^{\prime }$ and see a 340% increase in the LCS of compression elements. The LCS increases by an astounding 350% when $f_{u}$ is raised from 700 to 1150 MPa and $f_{c}^{\prime }$ is raised from 10 to 55 MPa. This demonstrates how the LCS of FRP-RC concrete compression elements is similarly affected by increasing the tensile stress of the FRP bars and the compression stress of the concrete.

### 5.2 Effectiveness of reinforcement ratio ($\rho_{l}$)

Fig. 14 further illustrates how the reinforcement ratio affects things. There have been several levels at which this metric has been studied: 5.35%, 4.86%, 4.38%, 3.89%, 3.41%, 2.92%, 2.43%, 1.94%, 1.46%, and 0.97%. The LCS improves by 35% when $f_{c}^{\prime }$ rises from 10 to 55 MPa, which is equivalent to the increase in ${\;\rho }_{l}$ from 0.97% to 5.35%. As a result, raising $f_{u}$ from 700 to 1150 MPa and increasing ${\;\rho }_{l}$ by 0.6% adds to an overall increase of 0.6%. Moreover, increasing D from 150 to 375 mm causes a 150% increase in LCS, while at the same time, ${\;\rho }_{l}$ rises from 0.97% to 5.35%.

#### 5.2.1 Effectiveness of stress of FRP bars ($f_{u}$)

The impact of varying $f_{u}$ on the LCS is depicted in Fig. 14. We investigated a range of $f_{u}$ values with increments of 50 MPa, ranging from 700 to 1150 MPa. When $\rho_{l}$ grew from 0.97% to 5.35%, the increase in axial compression stress caused by the increase in $f_{u}$ from 700 MPa to 1150 MPa was only 0.6%. Remarkably, raising D from 150 to 375 mm resulted in a 150% rise. Likewise, raising $f_{u}$ from 700 MPa to 1150 MPa resulted in a 350% increase in axial compression stress when raising $f_{c}^{\prime }$ from 10 MPa to 55 MPa. Furthermore, it was shown that the concrete stress and compression member area had a significant effect on the axial compression stress, especially when compared to other factors

## 6 Theoretical analysis

### 6.1 Database

The database of 600 GRC compression elements generated by the parametric study (discussed in the previous section) performed using the suggested FEA equation was used to suggest a new empirical equation that was authenticated using the test data of GRC compression elements examined in the present study. The estimates of the currently suggested equation were compared and validated using the predictions of previously suggested equations for the LCS of GRC compression elements tested in the present work. For axial reinforcement, GFRP bars were used in all of the elements, whereas GFRP hoops were used for crosswise confinement. The established database contains the tensile stress of FRP bars ($f_{u}$), the CS of concrete ($f_{c}^{,}$), modulus of elasticity of FRPs ($E_{f}$), peak tensile strain of FRPs ($\varepsilon_{u}$), crosswise reinforcement ratio ($\rho_{t}$), reinforcement ratio of FRPs ($\rho_{l}$), and the axial stress of FRP-RC compression elements ($P_{n})$. Table 9 contains the details of all data obtained from the parametric investigation.

**Table 9:** Details of constructed database (COV shows the coefficient of variance and St. Dev shows the standard deviation).

Parameter	*D* (mm)	*H* (mm)	$A_{g}$ (mm^2^)	$f_{c}^{\prime }$ (MPa)	$f_{u}$ (MPa)	$E_{f}$ (GPa)	$\varepsilon_{u}$*(%)*	$\rho_{l}$*(%)*	$\rho_{t}$*(%)*	$A_{f}$ (mm^2^)	$P_{n}$ (kN)
Minimum	150	300	39000	10	700	50	1.5	1	1.5	506	245
Largest	375	300	97500	55	1150	50	1.5	292	1.5	2785	2792
Mean	260	300	60125	31.3	837	50	1.5	6.9	1.5	1076	1309
St. Dev	45	0	15514	10.2	143	0	0	37	0	768	498
COV	5.76	–	3.9	3.1	5.8	–	–	0.2	–	1.4	2.6

### 6.2 Assessment of previous equations

Table 10 presents the existing equations for predicting the LCS of FRP-RC concrete compression elements that have been suggested in previous studies. Four different cases have been used for these equations depending on the axial involvement of the axial FRP rebars in the LCS of the compression elements and the axial capacity of the core to bear compression loading. The first case neglects the axial involvement of axial bars and the stress of core material [82,83] while the second case neglects the stress of the core but considers the axial involvement of axial bars depending on the stress of FRP bars [39,84,85]. Similarly, the third case neglects the stress of the core but considers the axial involvement of axial bars depending on the ultimate strain of concrete [[86], [87], [88], [89], [90], [91], [92]] while the fourth case considers the stress of the core and the axial involvement of axial bars [[93], [94], [95], [96]]. The second case considers the reduced tensile stress while the third case considers the strain of unconfined concrete at the ultimate loading stage. The studies present in the previous works [[93], [94], [95], [96]] proposing the fourth case consider both resistances provided by the axial GFRP rebars and the core material subjected to the condition that the axial GFRP rebars are sufficiently confined with the lateral reinforcement.

**Table 10:** The existing equation for LCS of FRP-RC concrete compression elements.

Research study	Suggested equation
**Case I:** Neglecting the axial involvement of axial bars and the stress of the core	
ACI 440.1R-15 [82]	$P_{n}=0.85f_{c}^{\prime }\left(A_{g}-A_{frp}\right)$
CAN/CSA S806-12 [83]	$P_{n}=\alpha_{1}f_{c}^{\prime }\left(A_{g}-A_{frp}\right)$; $\alpha_{1}{=0.85-0.0015f}_{c}^{\prime }\geq 0.67$
**Case II:** Neglecting the stress of the core but considering the axial involvement of axial bars depending on stress of FRP bars	
Tobbi et al. [39]	$P_{n}=0.85f_{c}^{\prime }\left(A_{g}-A_{frp}\right)+0.25f_{frp}A_{frp}$
Afifi et al. [84,85]	$P_{n}=0.85f_{c}^{\prime }\left(A_{g}-A_{frp}\right)+0.35f_{frp}A_{frp}$
**Case III:** Neglecting the stress of the core but considering the axial involvement of axial bars depending on the ultimate strain of concrete	
Xue et al. [86]	$P_{n}=0.85f_{c}^{\prime }\left(A_{g}-A_{frp}\right)+0.002E_{frp}A_{frp}$
Mohamed et al. [87]	$P_{n}=0.85f_{c}^{\prime }\left(A_{g}-A_{frp}\right)+0.002E_{frp}A_{frp}$
Tobbi et al. [88]	$P_{n}=0.85f_{c}^{\prime }\left(A_{g}-A_{frp}\right)+\varepsilon_{co}E_{frp}A_{frp}$
Youssef and Hadi [89]	$P_{n}=0.85f_{c}^{\prime }\left(A_{g}-A_{frp}\right)+\varepsilon_{co}E_{frp}A_{frp}$
Maranan et al. [90]	$P_{n}=0.9f_{c}^{\prime }\left(A_{g}-A_{frp}\right)+0.002E_{frp}A_{frp}$
Hadhood et al. [91]	$P_{n}=\alpha_{1}f_{c}^{\prime }\left(A_{g}-A_{frp}\right)+0.0035E_{frp}A_{frp}$; $\alpha_{1}{=0.85-0.0015f}_{c}^{\prime }\geq 0.67$
Hadhood et al. [92]	$P_{n}=0.85f_{c}^{\prime }\left(A_{g}-A_{frp}\right)+0.003E_{frp}A_{frp}$
**Case IV:** Considering the stress of the core and the axial involvement of axial bars	
Karim et al. [93]	$P_{n}=f_{cc}^{\prime }A_{cc}+f_{cc,cover}^{\prime }A_{cover}+\varepsilon_{cu}E_{frp}A_{frp}$
Hadi et al. [94]	$P_{n1}=0.85f_{c}^{\prime }\left(A_{g}-A_{frp}\right)+0.003E_{frp}A_{frp}$ for 1st peak $P_{n2}=0.85f_{cc}^{\prime }A_{cc}+\varepsilon_{cc}E_{frp}A_{frp}$ for 2nd peak
Pantelides et al. [95]	$P_{n}=0.85f_{cc}^{\prime }A_{cc}+0.003E_{frp}A_{frp}$
Hales et al. [96]	$P_{n}=f_{cc}^{\prime }A_{cc}+0.003E_{frp}A_{frp}$; when $f_{cc}^{\prime }A_{cc}\geq f_{c}^{\prime }\left(A_{g}-A_{frp}\right)$ $P_{n}=f_{c}^{\prime }\left(A_{g}-A_{frp}\right)+0.003E_{frp}A_{frp}$; when $f_{cc}^{\prime }A_{cc}<f_{c}^{\prime }\left(A_{g}-A_{frp}\right)$

Although various equations are available in the literature as discussed in Table 10, a few of them depending on their applications and importance in the previous studies were taken for the assessment of the developed database [41,95,[97], [98], [99], [100], [101], [102], [103], [104], [105], [106], [107]]. The mean squared error (MSE), coefficient of determination ($R^{2}$), root mean square error (RMSE), and mean absolute error (MAE) were used to evaluate these equations. These statistical coefficients are mathematically presented by Eq. (10), (11), (12), (13) [45,108]:

(10) $$R^{2}={\left(\frac{n\left(\sum_{i=1}^{n}x_{i}y_{i}\right)-\left(\sum_{i=1}^{n}x_{i}\right)\left(\sum_{i=1}^{n}y_{i}\right)}{\sqrt{\left[n\sum_{i=1}^{n}{x_{i}}^{2}-{\left(\sum_{i=1}^{n}x_{i}\right)}^{2}\right]\left[n\sum_{i=1}^{n}{y_{i}}^{2}-{\left(\sum_{i=1}^{n}y_{i}\right)}^{2}\right]}}\right)}^{2}$$

(11) $$MAE=\frac{1}{n}\sum_{i=1}^{n}\left|x_{i}-y_{i}\right|$$

(12) $$RMSE=\sqrt{\frac{1}{n}\sum_{i=1}^{n}{\left(x_{i}-y_{i}\right)}^{2}}$$

(13) $$MSE=\frac{1}{n}\sum_{i=1}^{n}{\left(x_{i}-y_{i}\right)}^{2}$$

**Fig. 15:**
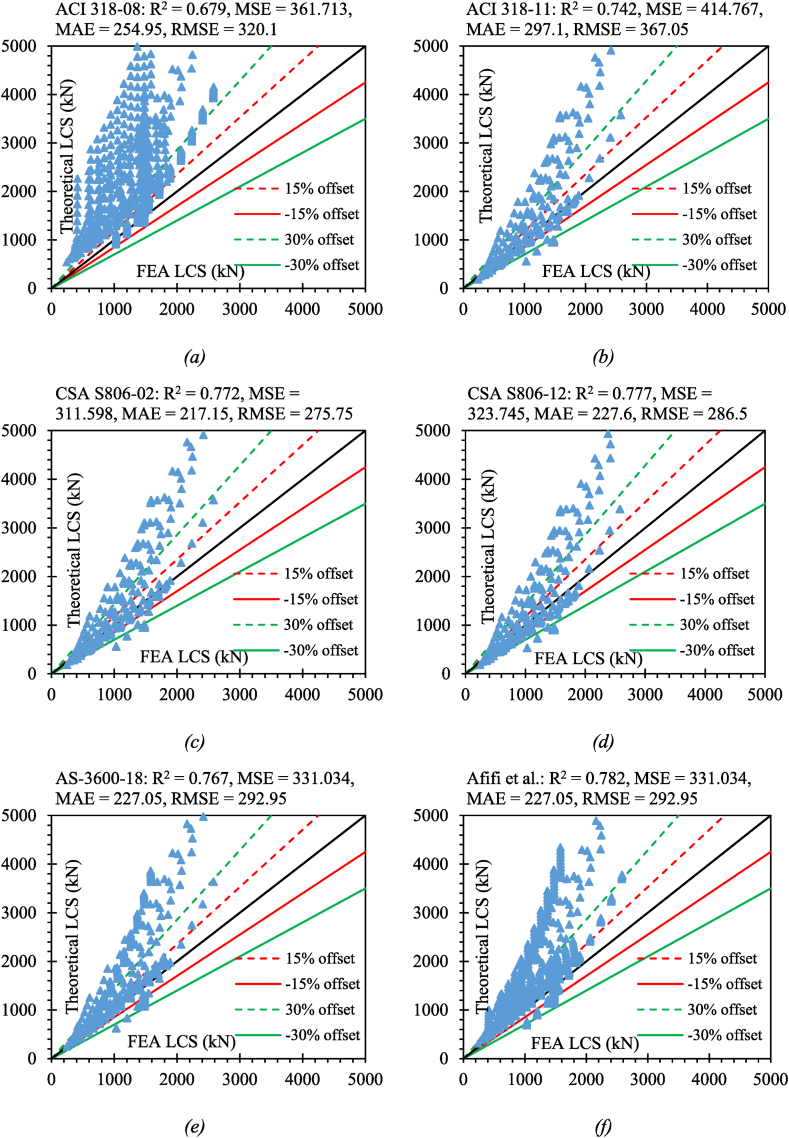

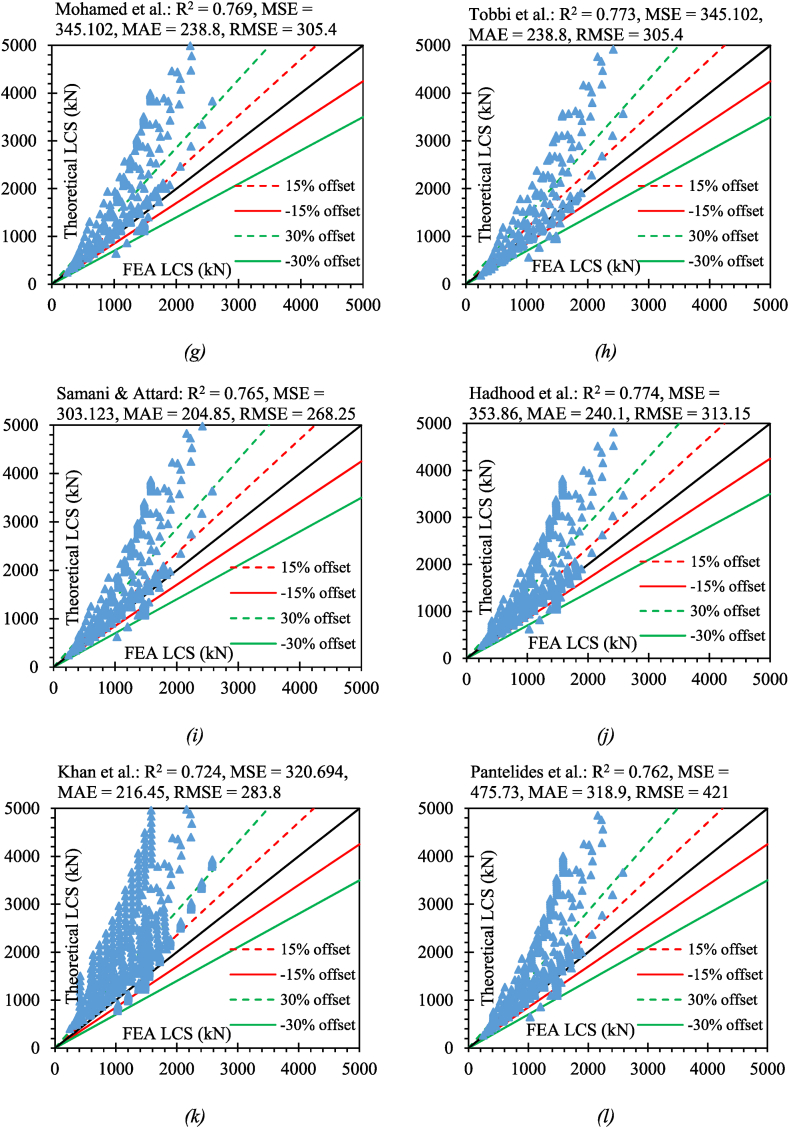
Efficacy of existing LCS equations on developed database for (*a*) ACI 318-08 (*b*) ACI 318-11 (*c*) CSA S806-02 (*d*) CSA S806-12 (*e*) AS-3600-18 (*f*) Afifi el al. (*g*) Mohamed et al. (*h*) Tobbi et al. (*i*) Samani & Attard (*j*) Hadhood et al. (*k*) Khan et al. (*l*) Pantelides et al.

Here, n denotes the total number of data points, $x_{i}$ denotes the LCS of GRC compression elements as determined by experiments, and $y_{i}$ denotes the LCS of GRC compression elements as determined by empirical equations. The evaluation of all empirical equations is shown in Fig. 15.

**Fig. 16:**
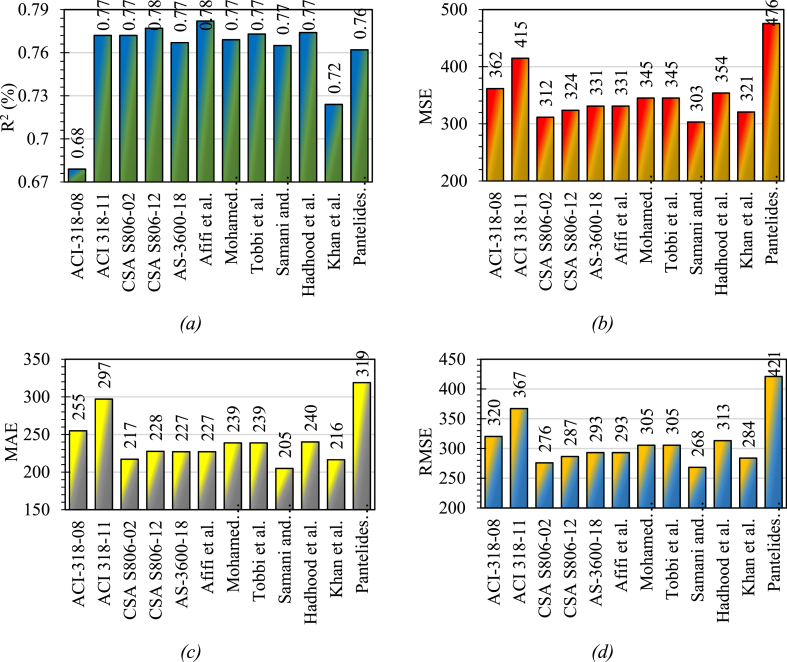
Statistical coefficients for the efficacy of previous equations on the developed database (*a*) R^2^ (*b*) MSE (*c*) MAE (*d*) RMSE.

The predictions of the previously suggested equations for GRC compression elements represented higher discrepancies because those equations depended on small data points that were suggested for a limited range of variables and specified compression elements. The presented equation in the study has been suggested by considering a large number of variables so that this equation can cover a wide range of FRP-RC compression elements. Fig. 16 shows the statistical coefficients for the efficacy of previous equations on the developed database. However, the efficacy of Afifi et al. [64] was higher than all other equations when we consider the coefficient of determination which is the most important statistical parameter to investigate the accuracy of a suggested equation. The equation suggested by CSA S806-12 [83] also showed a better response for this coefficient because this equation also considers the involvement of axial FRP bars while the equations suggested by the ACI code do not consider the axial input of FRP bars, therefore, they are giving relatively higher discrepancies. The equation suggested by Pantelides et al. [95] presented higher statistical errors. This is due to the reason that this equation was suggested depending on small noisy data considering higher values of coefficients.

#### 6.2.1 Confinement effectiveness

The impact of the glass-FRP ties providing lateral confinement improved the stress and ductility of GRC compression elements [64]. The confinement effect is amplified when the element crosses the largest LCS by reducing the dilatation of concrete generated by lateral pressure after the transmission of axial stress. The LCS of GRC compression elements has previously been calculated without considering the confinement effect of glass-FRP ties. The forecasts are understated because the impact of glass-FRP-confinement in the LCS of compression elements is ignored [109]. As a result, in this study, the confinement equations presented by Afifi et al. [64] were used to determine the stress and strain of FRP-confined concrete (Eq. (1) and (2)). The lateral confinement pressure ($f_{l}$) caused by glass-FRP ties can be represented by Eq. (14) [110].

(14) $$f_{l}=k_{e}\frac{{2f}_{fb}A_{tf}}{sd_{s}}$$

Where $k_{e}=A_{e}/A_{c}$ is the confining efficiency constant, $A_{c}$ is the core area, $A_{e}$ is the efficiently restrained core area, $f_{fb}$ is the bending stress of glass-FRP ties determined as 0.004 $E_{ft}$ [97], $A_{tf}$ is the area of glass-FRP ties, $d_{s}$ is the diameter of core, and s is the center to center distance between glass-FRP ties.

### 6.3 Model for LCS of GRC compression elements

The equation proposed by Afifi et al. [64] reported the highest accuracy with $R^{2}$ = 0.782. When $R^{2}$ is equal to one, it indicates that test outcomes and LCS forecasts are perfectly correlated. As a result, the current suggested equation's general form was assumed to be similar to that of Afifi et al. [64] to give higher accuracy. The role of FRP bars in affecting the axial stress of GRC compression elements was taken into account. The expression of the currently accepted equation is shown in Eq. (15). This equation is proposed depending on the 600 tested elements using the calibrated FEA equation during the parametric study. The equation has been suggested using the general regression analysis and curve fitting techniques to obtain the best fit to curve and improved values of various statistical coefficients where the core concrete was considered as the concrete confined with FRPs and the strain of concrete was considered equal to the strain of confined concrete.

(15) $$P_{n}=\alpha_{1}\left(A_{g}-A_{FRP}\right)\left\{f_{co}^{\prime }+4.547{f_{co}^{\prime }\left(\frac{f_{le}}{f_{co}^{\prime }}\right)}^{0.723}\right\}+\left\{\varepsilon_{co}^{\prime }+\varepsilon_{co}^{\prime }\left(\frac{0.024}{\varepsilon_{co}^{\prime }}\right){\left(\frac{f_{le}}{f_{co}^{\prime }}\right)}^{0.907}\right\}f_{FRP}A_{FRP}$$

In this equation, $\alpha_{1}$ represents a coefficient that will be determined using regression and curve-fitting analysis depending on a database of 600 elements in MATLAB, $A_{FRP}$ reflects the area of FRP axial bars, and $f_{FRP}$ illustrates the tensile stress of FRP bars. $A_{g}$ shows the gross area of the element, and $A_{FRP}$ represents the gross area of FRP axial bars. However to achieve the best match, the error functions were reduced to the greatest extent possible. The curve fitting method was used to gather the best fit for the test database, yielding a constant value of 0.80 for this coefficient. The following is the equation for LCS of GRC compression elements that take into account the lateral confinement effect given by the glass-FRP ties:

(16) $$P_{n}=0.80\times \left(A_{g}-A_{FRP}\right)\left\{f_{co}^{\prime }+4.547{f_{co}^{\prime }\left(\frac{f_{le}}{f_{co}^{\prime }}\right)}^{0.723}\right\}+\left\{\varepsilon_{co}^{\prime }+\varepsilon_{co}^{\prime }\left(\frac{0.024}{\varepsilon_{co}^{\prime }}\right){\left(\frac{f_{le}}{f_{co}^{\prime }}\right)}^{0.907}\right\}f_{FRP}A_{FRP}$$

**Fig. 17:**
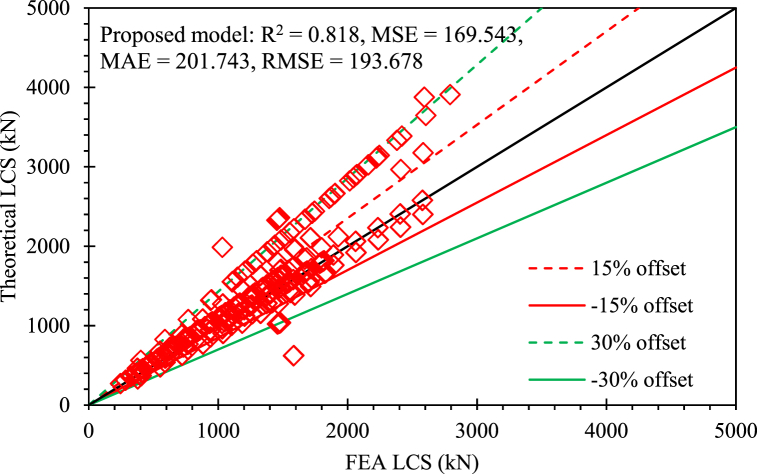
Efficacy of the suggested equation.

The newly suggested equation was more accurate than the preceding equations. Concerning the validity of the suggested equation, this is valid for the conditions: the variable $f_{co}^{\prime }$ should have a range of 10–55 MPa, The reinforcement ratio of axial bars should have a range of 0.97–5.35%, and the parameter $f_{FRP}$ should have a range of 700–1150 MPa, and the ultimate strain of FRP reinforcement should have a value of 1.5%. The suggested equation (Eq. (16)) functioned well with the values of statistical indices as $R^{2}$ = 0.818, MSE = 169.543, MAE = 201.743, and RMSE = 193.678. Fig. 17 shows the efficacy of the suggested empirical equation. 5 points were overestimated by the suggested theoretical equation by more than 30% while 45 points showed an error of more than 15% in overestimation. Similarly, 4 points were underestimated by the suggested theoretical equation by more than 30% while 18 points showed an error of less than 15% in underestimation. Therefore, the database should be improved by removing the noisy data points to obtain higher accuracy of the suggested equation.

**Fig. 18:**
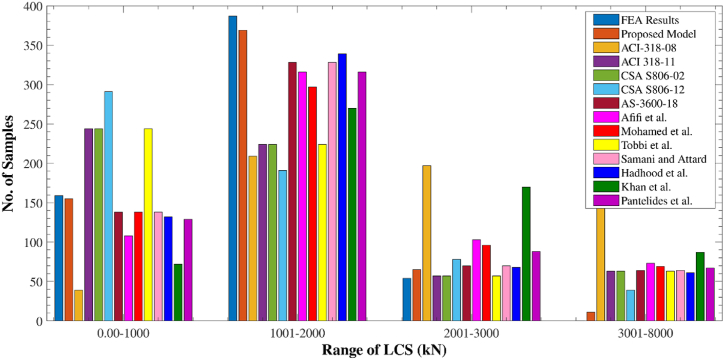
Distribution of LCS of elements for various equations.

**Fig. 19:**
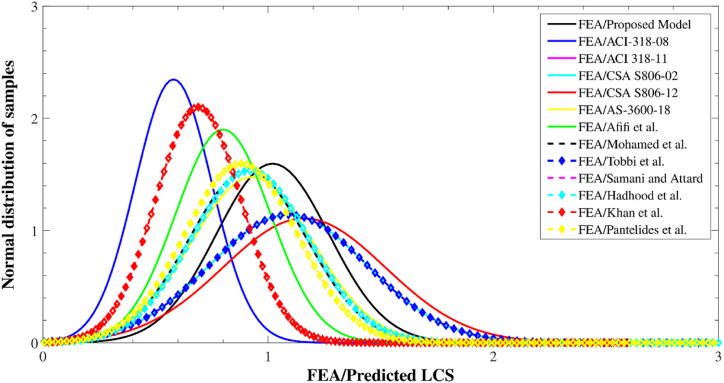
Normal distribution of test outcomes to predictions for various equations.

Fig. 18 shows the spread of predictions as well as test outcomes for the LCS of GRC compression elements. There were 159 different test values for LCS in the database, ranging from 0 to 1000 kN. The recommended equation provided 155 values in this range. Meanwhile, the 1001–2000 kN range had 387 testing and 369 estimated values, the 2001–3000 kN range had 54 testing and 65 estimated values, and the 3001–8000 kN range had 0 testing and 11 estimated values. These figures further demonstrate that the proposed model accurately predicted the axial stress of GRC compression elements. The axial stress of GRC compression elements is shown in Fig. 19 as regularly distributed testing and estimated values. With only a 3% deviation from unity, the suggested formula worked well for average standardized values of ratios of empirical LCS to expected stress. An overestimate of 76% was seen for the largest estimate of ACI-318-08 [111]. The deviation could be because the ACI-318-08 [111] suggested equation is for traditional steel bars, while this equation is only used for relative analysis. Furthermore, the discrepancies for the Khan et al. [106], Afifi et al. [41], and CSA S806-12 [83] equations were 59%, 27%, and 24%, respectively.

**Fig. 20:**
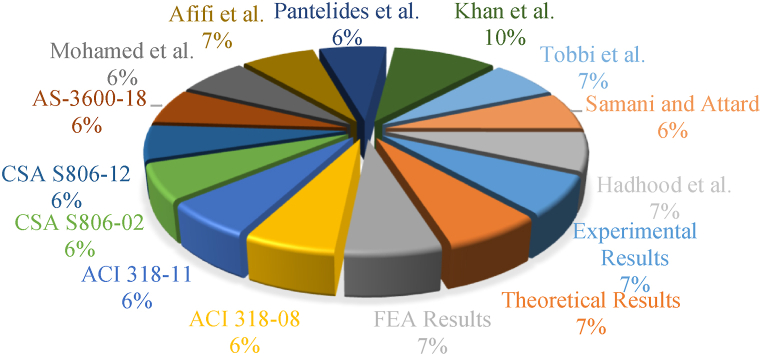
Comparison of predictions of various equations for the LCS of GRC elements.

The suggested equation accurately represented the LCS of GRC compression elements, as shown in Fig. 20. For GRC compression elements, the recently suggested equation for the LCS of compression elements presented a mean percent inaccuracy of 2.55%. The average percent difference between the suggested empirical equation's predictions and FEM's predictions was 1.23%. The LCS of GRC compression elements had bigger variances than in previous equations. Thus, the suggested empirical equations' predictions thoroughly validate their applicability and accuracy for reasonably apprehending the LCS of GRC compression elements when glass-FRP crosswise and axial bars are considered.

## 7 Conclusions

The present study has endeavored to investigate the structural behavior of glass-FRP-RC rubberized concrete (GRC) compression elements by experiments, FEA, and theoretical analysis. The following are some important points taken from this research.

1. The failure was mostly detected in the upper portion and the middle of the compression elements. The compression elements were damaged due to a crack in the axial bars and a rupture in the glass-FRP ties. The controlling failure indicates the amplified deformability efficacy of rubberized concrete. The main reason behind the failure of rubberized concrete is the micro-cracking within the concrete which outcomes in the controlled failure without a sudden crushing like normal concrete showing an abrupt failure.
2. Due to well-restrained axial bars and improved confining action of the core area to absorb more energy, the compression elements with lower stirrup spaces exhibited greater ductility indices. Furthermore, the addition of rubberized aggregates presented an improved ductility of GRC compression elements.
3. The LCS of GRC compression elements was improved due to the lessening in stirrup spaces. For GRC compression elements with eight glass-FRP bars, lowering the stirrup spaces from 150 mm to 75 mm resulted in a 4% increase in percentage. For GRC compression elements with eight glass-FRP bars, a percentage drop of 12% was recorded when the stirrup spaces were reduced from 225 mm to 150 mm.
4. Increasing the number of glass-FRP axial bars from eight to ten enhanced the LCS of GRC compression elements; however, employing ten axial bars reduced it due to the brittle behavior of over-reinforced elements.
5. Using the updated CDP equation, the suggested FEM accurately anticipated the complex behavior of GRC. The recommended FEM for the LCS and related axial strain of examined elements had average percent errors of 3.58% and 4.01%, respectively. ABAQUS properly replicated the failure behavior of the elements.
6. The parametric analysis of 600 elements revealed that the LCS of concrete and the cross-sectional size of a concrete compression member had a considerable impact on the LCS of these elements. When the cross-sectional area of the compression member was amplified to 1.5 times, the LCS amplified by 1040%. Because of increasing the concrete stress to 4.5 times, the LCS of compression elements amplified by 350%. The LCS was found to be unaffected by the reinforcing ratio of FRP bars or the tensile stress of FRPs.
7. The recommended theoretical equation for the LCS of GRC compression elements worked well for the test findings of the analyzed elements with $R^{2}$ = 0.818, MSE = 169.543, MAE = 201.743, and RMSE = 193.678. The average differences between the suggested FEA and empirical equations from the test outcomes for the LCS of elements tested in the current investigation were 2.52% and 2.55%, respectively. It can be said that the predictions for the LCS, failure modes, and ductility of the glass-FRP-RC rubberized concrete compression elements using the suggested equations are excellent.

## Data availability statement

Data will be available upon request from the corresponding author.

## CRediT authorship contribution statement

**Ali Raza:** Conceptualization, Data curation, Project administration, Resources, Writing – original draft. **Khaled Mohamed Elhadi:** Data curation, Funding acquisition, Software, Visualization, Writing – review & editing. **Muhammad Abid:** Data curation, Formal analysis, Validation, Visualization, Writing – review & editing. **Ahmed Farouk Deifalla:** Data curation, Formal analysis, Resources, Software, Writing – review & editing. **Muhammad Sohail Jameel:** Data curation, Formal analysis, Software, Writing – review & editing. **Yasser Alashker:** Conceptualization, Methodology, Resources, Supervision, Writing – original draft.

## Declaration of competing interest

The authors declare that they have no known competing financial interests or personal relationships that could have appeared to influence the work reported in this paper.
